# Vaccinomics and Personalized Vaccinology: Is Science Leading Us Toward a New Path of Directed Vaccine Development and Discovery?

**DOI:** 10.1371/journal.ppat.1002344

**Published:** 2011-12-29

**Authors:** Gregory A. Poland, Richard B. Kennedy, Inna G. Ovsyannikova

**Affiliations:** Mayo Vaccine Research Group, Department of Medicine, Mayo Clinic College of Medicine, Mayo Foundation, Rochester, Minnesota, United States of America; The Fox Chase Cancer Center, United States of America

## Abstract

As is apparent in many fields of science and medicine, the new biology, and particularly new high-throughput genetic sequencing and transcriptomic and epigenetic technologies, are radically altering our understanding and views of science. In this article, we make the case that while mostly ignored thus far in the vaccine field, these changes will revolutionize vaccinology from development to manufacture to administration. Such advances will address a current major barrier in vaccinology—that of empiric vaccine discovery and development, and the subsequent low yield of viable vaccine candidates, particularly for hyper-variable viruses. While our laboratory's data and thinking (and hence also for this paper) has been directed toward viruses and viral vaccines, generalization to other pathogens and disease entities (i.e., anti-cancer vaccines) may be appropriate.

## Introduction

The goal in vaccinology is to discover, develop, and deploy highly immunogenic and safe vaccines that protect against infectious and non-infectious (i.e., cancers) diseases in essentially 100% of the population. While admirable, such a goal, to date, fails because of both pathogen and host variability. For hyper-variable viral pathogens like HIV, HCV, rhinovirus, and others, we have been unable to discover and develop highly immunogenic and protective vaccine candidates. This is true too for other highly complex pathogens such as bacteria (i.e., tuberculosis) and parasites (i.e., malaria). Host variability is evident in the multiplicity of immune response genes that encode >10^12^ products necessary for generating immune responses (i.e., antibodies, T cell receptors [TCRs], etc.), and the estimated diversity of human leukocyte antigen (HLA) haplotypes (estimated at >10^13^), allowing humans an almost limitless immune response capability [1].

Thus, both pathogen *and* host variability barriers make it difficult to induce protective immune responses to vaccine antigens in 100% of the population—at least for most of the pathogens of interest for vaccine public health needs such as HIV, HBV, HCV, measles, influenza, and others.

## Current Vaccine Development

We propose that an additional approach to this dilemma resides in changing the paradigm and conceptual framework through which we develop new vaccines. For example, from the 1700s through the late 1990s, vaccine development was primarily characterized by an empiric “isolate – inactivate/attenuate – inject” approach. While successful in developing most of the vaccines we use today, it fails in the face of hyper-variable and highly complex pathogens and is an approach now limited by a lack of innovation, a predominant single mode of administration (injection), and a lack of directed adjuvants to overcome poor immunogenicity of the identified antigen. From a policy viewpoint, today's vaccines are administered to everyone at the same dose (“one dose fits all”) as a public health approach that assumes that everybody is at risk for every pathogen with equally devastating risks of complications. Too, our past and current approach to vaccines is prophylactic only (we have no therapeutic vaccines), is overwhelmingly aimed at childhood diseases (ignoring demographic trends of aging populations in every developed economy), and at least in the US, is exclusively a private sector, big Pharma manufacturing approach.

## Vaccinomics and Directed Vaccine Development

Our laboratory has advocated for a new approach to vaccine discovery characterized as a “discover – validate – characterize – deploy” paradigm based on the foundations of vaccinomics and personalized vaccinology [2]–[4]. This approach moves away from a focus on the smaller details of immune function and advocates pursuing an understanding of the immune system as a whole in order to improve and expand upon empirical vaccine science. Furthermore, the approach is personalized in that it emphasizes a tiered risk and vaccination approach for new vaccines, multiple avenues of vaccine administration that take advantage of new findings (e.g., in mucosal immunology allowing for oral, transcutaneous, depot, and mucosal delivery), multiple highly specific vaccine adjuvants, directed vaccine development using systems biology and computational approaches, and private, public, and academic partnerships in the development of new vaccine candidates. An initial aspect of this new approach is the concept of reverse vaccinology, which uses sophisticated computer analysis of genomic data to characterize pathogen antigens and eliminate those with human homology. This is followed by careful screening of the remaining antigens for immunogenicity and eventual use in new vaccine products [5], [6]. For example, reverse immunology was used to create a recombinant protein containing nine different Th epitopes that has been used to enhance the hemophilus influenza type b oligosaccharide vaccine [7]. A large number of reverse immunology studies have focused on the characterization of T cell responses to vaccinia virus and have identified hundreds of CD4 and CD8 T cell epitopes. Other studies have carefully examined the vaccinia transciptome. [8]. Vaccinomics seeks to better and more fully integrate these findings, correlating humoral and cellular immune measures with transcriptomic, genomic, and proteomic data to gain a greater understanding of viral immunity. One such integrated study has uncovered complex interactions between CD4, CD8, and humoral responses to vaccinia virus [9].

**Figure 1:**
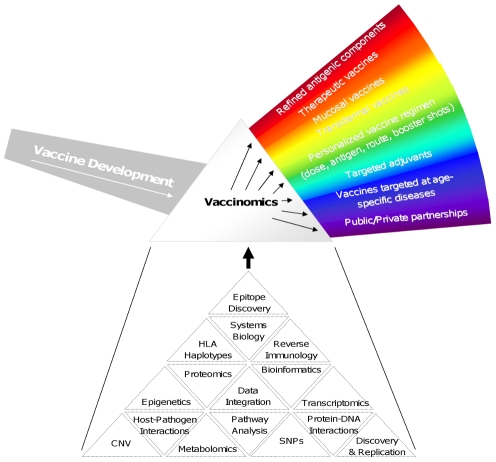
New approach to vaccine discovery and development. Figure 1 illustrates the differences between the one-size-fits-all approach of empiric vaccine development with a more directed and personal approach that relies upon vaccinomics and high-dimensional “omics” technologies. By analogy, empiric vaccine development represents the undifferentiated light entering the prism from the left. Individual aspects of directed vaccine development can be seen when viewed through the prism of vaccinomics. Several examples of these components are illustrated in the rainbow on the right side of the figure. These aspects may or may not be appropriate for all vaccines and are used here to illustrate the wide range of possibilities that a “discover – validate – characterize – deploy” approach allows one to independently investigate, optimize, and fully utilize. Below the vaccinomics prism are listed some examples (by no means complete or definitive) representing a range of potential components that can be assembled into a comprehensive, systems-level examination of infection/vaccination of a given pathogen. Please refer to the text for examples of how different components might be used in the development of specific vaccines.

Figure 1 outlines some of the important features that might be employed by a vaccinomics approach. It should be noted that not all of these features may be needed for a given vaccine, and those features that are needed may be prioritized differently depending on the unique constraints imposed by the disease, vaccine product, and/or population to be protected. For example, cancer vaccines have benefited greatly from advanced bioinformatics, reverse immunology, and epitope discovery to develop very personalized products. On the other hand, new vaccines against leishmaniasis or Japanese encephalitis for use in developing countries where distribution and inoculation are handled by public/private organizations with access to at-risk populations and familiarity with the local culture and society may require the development of a stable product not requiring a cold chain and transdermal application such that advanced training is not required for administration.

The need for and importance of new advances in vaccinology such as those above may not be apparent to all. Above and beyond the obvious value in decreasing (or eliminating in the case of smallpox, and hopefully soon polio and measles) morbidity and mortality due to infectious diseases, economic benefits accrue to healthy populations, national security may be enhanced, bioweapons development countered, and new insights into vaccine immunology generalized into other fields. As a result of these compelling arguments for vaccines, we deliver a series of vaccines to every human being on earth, multiple times over a lifetime. The importance of this is that there *is nothing comparable in medicine that so touches every single human being.* Indeed, this public health approach toward vaccine use has contributed to a doubling of the lifespan in the US over the last century by the control of infectious diseases, and the supportive role played by vaccines. But, as mentioned, it has been a one-size-fits-all approach. In the 21st century we may now ask, is such an approach, for our time and age, informed by science?

A variety of factors impact the heterogeneity and inter-individual variations in vaccine-induced immune responses. These include factors such as gender [10], age [11], ethnicity [12], vaccine dose [13], vaccine storage/cold chain [14], immune system function/integrity [15], size (body mass index [BMI]) [16], smoking [17], and others. Logically, genetics play an important—and defining—role in vaccine response. We increasingly understand the role of *genetic* causes of heterogeneity in treatment effects with *drugs,* but similar work in the field of vaccinology has lagged. One investigator has observed, “Just as pharmacogenetics has suggested ways of designing drugs to minimize population variability, understanding mechanisms of *immunogenetic* variation may lead to new vaccines designed to minimize immunogenetically based failure” [18]. This naturally leads to such questions as, “why do immune responses to biologics/vaccines vary among healthy individuals? “And what explains this heterogeneity?” “Could the answers to these questions be leveraged in reverse engineering new vaccine candidates?”

## The Immune Response Network Theory

While we readily accept that genetic variation in TCR genes, antibody genes, and HLA loci all contribute to the differential ability of the host to respond to pathogens, these are not the only genes that impact vaccine immunity. Host genetic influences on inter-individual variability can also occur as a result of polymorphisms in genes involved in the generation of the immune response, including viral receptors, Toll-like and other pattern recognition receptors, signaling molecules, cytokine and cytokine receptor genes, Gm/Km genes, perforin and granzymes, and death receptors, as well as many others. In recognizing this, our laboratory developed the “Immune Response Network Theory,” which states that the response to a vaccine is “the cumulative result of interactions driven by a host of genes and their interactions, and is theoretically predictable” [19], [20]. This theory is different than Jerne's idiotype network theory stating that the antigen recognition site of one antibody can in turn serve as an antigen stimulating the production of anti-idiotype antibodies, and that these networks of antibodies/anti-idiotypic antibodies serve to positively and negatively regulate immune function [21]. “The basic genetic elements of the immune response network includes genes activating/suppressing immune responses, the dominance profile of a given gene or polymorphism, epigenetic modifications of genes, the influence of signaling genes, innate response genes, gene-gene interactions, and genes for other host response factors” [2]. Understanding the complex interplay of these networks and pathways as a coherent system allows one to build predictive models, anticipate possible side effects, and observe synergistic outcomes that cannot be foreseen with narrowly focused studies concentrating on single genes or proteins or even single cell types. Understanding the key initial events in the immune response to pathogen infection allows us to identify viral ligands responsible for cell binding and entry, innate receptors responsible for pathogen detection, innate pathways mediating protective responses specific for a given pathogen, host pathways usurped by viral machinery, and pathogen epitopes targeted by T and B cells, and the interplay between T helper lymphocytes and B cells or cytotoxic T cells necessary for optimal humoral and cell-mediated responses. In turn, this information allows for the identification of adjuvants stimulating the appropriate innate receptors and antiviral pathways, attenuation strategies for the pathogen of interest, the appropriate selection of viral epitopes for subunit vaccines, vaccine products that omit the viral proteins responsible for pathogen-induced damage and suppress the host pathways responsible for immunopathology, the effects that different routes of administration have on the immune response, and the appropriate dose/route/timing of immunizations to properly elicit strong immune memory.

As we have noted, the mechanisms for differential gene-based effects can include “differential binding, processing, and expression/presentation of antigenic peptides, a differential range of presented peptides (genetic restriction), altered secretion patterns (cytokines), altered transcription of important genes (signaling molecules) and gene products, altered binding of virus/antigens by membrane-based receptors (TLR, other), differential receptor function, expression, affinities, epigenetics, and of course, others” [22]. Further, our laboratory developed the term “vaccinomics” to encompass the integration of a systems biology approach with immunogenetics, immunogenomics, immune profiling, and functional SNP studies in order to understand and predict vaccine-induced immune responses. Using these concepts we have predicted “a new golden era of personalized *Predictive Vaccinology*” whereby we abandon a “one size and dose fits all vaccine approach,” predict whether to give a vaccine based on likelihood of response (and perhaps need), predict the likelihood of a significant adverse event to a vaccine, predict the number of doses likely to be needed to induce a response to a vaccine (HBV, HPV, measles examples), and design/develop new vaccines at the individual, gender-specific, race-specific, or sub-population levels for groups with identifiable and specific genetic restrictions [3], [4], [23].

## Genetic Control of Measle Vaccine Response

**Figure 2:**
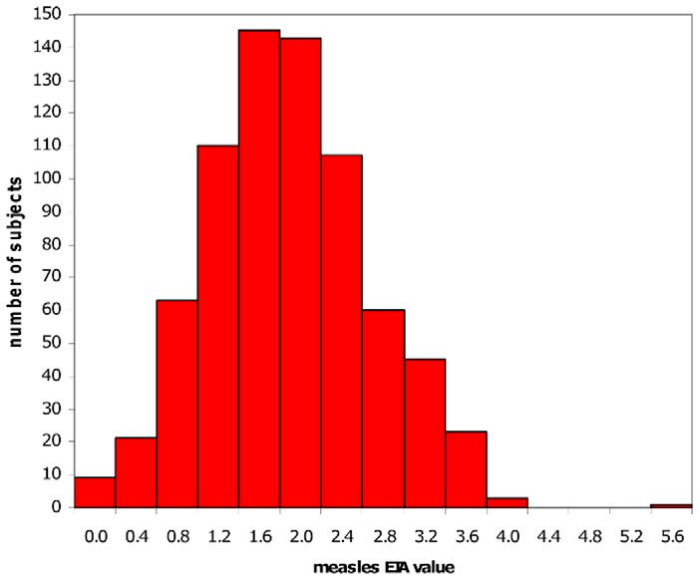
Distribution of measles vaccine–induced antibody levels. This graph represents the distribution of antibody levels determined by an EIA assay on healthy grade-school children immunized with a single dose of MMR-II vaccine. The inter-individual variation in antibody levels among this healthy cohort illustrates the importance of determining the mechanisms for heterogeneity in vaccine response.

As examples, we have focused our work in vaccinomics on the study of measles, rubella, smallpox, and influenza vaccines. In order to understand the role of genetic (host) variation in inter-individual vaccine-specific immune responses, we began by performing twin studies (*n*  =  100 twins) to separate environmental and genetic influences, to determine the influence of genetic factors relating to variability in immune response, to determine the proportion of variation attributable to specific genes, and to determine heritability (the ratio of genetic variance to total variance) [24]. In this study we determined that the heritability of measles vaccine was 89% (*p* < 0.0001) [25]. We next studied a cohort of healthy schoolchildren, all immunized with one dose of MMR-II (medical record documentation), with no circulating measles in the community since 1980 (the earliest year of birth) [26], [27]. The results are noted in Figure 2, which demonstrates the diversity of inter-individual antibody response to vaccine among otherwise healthy schoolchildren.

Among the above individuals who were vaccine non-responders, we re-immunized, and repeated antibody testing ≥ 6 weeks later. One hundred and six children (81.5%) became seropositive, and 24 (18.5%) remained seronegative [28]. We then re-examined our candidate gene associations in individuals who had received 2 doses of measles vaccine, and our previously detectable class I and II HLA effects were no longer detectable except for B*4403 [22]—a similar finding in studies of hepatitis B vaccine non-responders. Similarly, two doses of measles vaccine appeared to overcome HLA homozygosity associations with lower measles-specific antibody and cytokine levels detected following one dose of MMR vaccine [29], [30]. These findings illustrate that there is HLA-restricted recognition of measles virus epitopes with detectable impacts on immunity, and that through an as yet unclear mechanism, additional doses of vaccine may help to overcome this genetic restriction, including non-responsiveness to measles vaccine.

Our population-based MMR vaccine studies also determined that host gene polymorphisms are associated with measurable inter-individual variations in measles vaccine–induced immunity. Examples of such gene SNPs include HLA, measles virus binding CD46 and SLAM receptors, cytokine and vitamin receptors, as well as innate antiviral effector genes, including Toll-like receptors (TLRs) and their signaling genes, which play a significant role in contributing to variations in the immunity to measles due to genetic polymorphisms [31]–[34]. The adaptive immune response after measles vaccination is influenced in part by HLA gene polymorphisms. In fact, the occurrence or lack of specific HLA alleles and haplotypes (or supertypes) may significantly influence both humoral and cellular immune responses to a vaccine. Furthermore, our studies have demonstrated that genetic polymorphisms in measles virus receptor genes, pattern recognition receptor genes, genes controlling innate antiviral responses, and cytokines and cytokine receptor genes are associated with variations in measles vaccine–induced immune outcomes.

### Associations with Measles-Specific Humoral Immunity

We found several HLA alleles (B*3503, DRB1*0701, and DQA1*0201) and haplotypes (A*29-C*16-B*44 and DRB1*15/15-DQB1*06-DPB1*03) with associations with measles-specific neutralizing antibody levels in two independent population-based studies. Individual genetic variants in the CD46 (rs11118580 and rs2724384) and SLAM (rs164288) genes that appear to modulate antibody responses to measles vaccine were also identified [33]. Increased carriage of major allele variants for coding SNPs in the TLR2 (rs3804100) and TLR4 (rs5030710) genes were associated with a dose-related increase and a dose-related decrease in measles antibody levels, respectively [34]. Recently, we also replicated a previously discovered association of a functional IL12B genetic variant rs3212227 with inter-individual variations in measles-specific antibody levels [35]. Genetic variants within the RIG-I gene, including a coding polymorphism (rs3205166), were associated as single-SNPs and in haplotype-level analysis, with measles antibody variations [36].

### Associations with Measles-Specific Cellular Immunity

In a separate study we successfully replicated associations with two of the above mentioned measles virus receptor SNPs (CD46 rs2724384 and SLAM rs164288) and variations in measles antibody and IFN-γ Elispot responses, respectively [37]. A replicated CD46 polymorphism (rs2724384) also demonstrated associations with measles-specific IL-6 (*p*  =  0.02), IFN-α (*p*  =  0.007), and TNF-α (*p*  =  0.0007) responses. Two previously reported promoter IL10 and IL2 SNPs (rs1800890 and rs2069762) demonstrated associations with measles-specific cellular response (*p* < 0.03) [38]. A different polymorphism (rs11265452) in the SLAM gene previously associated with measles antibody levels (*p*  =  0.04) exhibited a significant association with measles-specific IL-10 production (*p*  =  0.0008) [37]. Understanding the functional or mechanistic consequences of genetic variations such as those above on immune-response variations could assist in directing new vaccine design, and allows us to generate and test new hypotheses applicable to developing new measles vaccine candidates.

## Additional Examples of Directed Vaccine Development

Taking these concepts further, hepatitis B vaccine serves as a useful example. Both HLA polymorphisms and cytokine SNPs have been found to be associated with hepatitis B vaccine non-response [39]. This information could be utilized by developing a candidate vaccine that included both cytokine adjuvants to overcome genetic restriction, and a peptide “cocktail” that could circumvent known immunogenetic restrictions, and investigators have begun such development [40], [41]. Similarly, we have previously reported a SNP in the SLAM receptor gene associated with a 4-fold decrease in measles antibody levels [33]. While mechanistic studies are ongoing, it is logical that this SNP may interfere with the ability of the measles vaccine virus to bind to its receptor, and thereby perturb the development of a protective immune response. One could imagine a candidate vaccine virus designed to allow binding regardless of the presence or absence of such a receptor polymorphism. Such a vaccinomics approach could result in a candidate vaccine that leads to protective immune responses regardless of the presence of such a polymorphism. As a further example, such an approach led to the identification of the CCR5 deletion mutation in the coding region of the CCR5 HIV receptor. This information can be utilized in the development of novel therapeutic drugs and vaccines [42]. For example, a subunit vaccine containing the CCR5 binding determinants of gp120 could be created to facilitate the formation of viral neutralizing antibody responses. In addition, adjuvants such as CpG or MPL-A could differentially activate TLRs to circumvent restrictions in other receptors [43].

Other limitations in the development of new vaccines for measles and other infectious pathogens include a lack of understanding of molecular mechanisms of vaccine-induced adaptive immunity. While we understand that viral peptides are processed and presented in the context of class I and II HLA molecules, this has generally not informed the specific design of new vaccine candidates. Our laboratory has used this information to successfully identify 13 novel naturally processed class II HLA-DRB1*0301 measles virus peptides [44], [45]. The development of a high performance mass spectrometry analytic approach also allowed us to identify 116 naturally processed and presented class I (A*0201, B*1501 and C*03) peptides derived from vaccinia virus [46], [47]. Recently, we also isolated 17 naturally processed avian influenza H5N1 peptides from the class I A*0201 peptide binding grove (P. Tosh, I. Ovsyannikova, G. Poland, unpublished data). Data on specific immunogenic peptides (and adjuvants) such as these become important in the design of future vaccines to combat infectious diseases, including measles, influenza, smallpox, and other pathogens [48].

## Conclusion

Our laboratory has used the live, attenuated measles, mumps, rubella, and vaccinia vaccine viruses as models for our work and development of the vaccinomics approach. After two decades of work with measles vaccine virus we have determined that:

- Almost 90% of measles vaccine response heterogeneity is explainable genetically
- Polymorphisms of specific immune response genes significantly influence measles vaccine–induced immunity
- Vaccine-induced immune responses can be profiled (and in the near future predicted)
- Naturally processed and presented immunogenic peptides can be identified and sequenced, and represent a novel method of vaccine candidate discovery

The next step in the development of vaccinomics is to understand immune “signature profiles” from a systems biology perspective in order to develop vaccine response “markers” in support of personalized vaccinology, and to inform new vaccine development. An excellent example of this concept is the identification of a gene signature including C1QB and EIF2AK4, which correlated with and predicted CD8+ T cell responses to the yellow fever vaccine with a high degree of accuracy [49]. The authors also identified a separate predictive signature of neutralizing antibody response that included the B cell growth factor TNFRS17. Yet another example of predictive immune profiling has been demonstrated for influenza vaccination. The expression levels of CAMKIV (a calmodulin-dependent protein kinase involved in neural functions as well as stem cell maintenance and T cell development) at day 3 following vaccination with TIV is inversely correlated with antibody titers at the peak of the immune response [50]. The development of these predictive signatures provides significant insights into the generation of vaccine-induced immune responses, and may serve as useful biomarkers for the testing of novel vaccine candidates. The knowledge gained from these immune-profiling studies may indicate appropriate adjuvants or routes of administration that can be coupled with mass-spectrometry approaches to isolating and identifying highly immunogenic viral peptides, allow for peptide-based vaccine development (an area of vaccine research that currently suffers from poor immunogenicity), and allow us to anticipate novel, directed development (rather than an empiric approach) of a plethora of new candidate vaccines informed by genotype:phenotype associations, the role of epigenetics and complementarity, and other future advances.

We believe that the future of vaccine development, utilizing the tools of vaccinomics and predictive vaccinology, is such that the science will move us to abandon a “one size and dose fits all empiric vaccine approach,” predict vaccine response and the possibility of a significant adverse response to a vaccine, predict the number of doses likely to be needed to induce a response to a vaccine, and direct us toward a science-based directed design/develop paradigm for novel vaccine candidates. In turn, abandoning the empiric approach of vaccine development, and moving toward a new paradigm of “discover – validate – characterize – deploy” is likely to hold promise in the development of vaccine candidates for hyper-variable pathogens, and overcome the current one-size-fits-all approach that leads to substantial inter-individual vaccine responses, vaccine non-response, increased costs, and substantial barriers to the development of novel vaccine candidates.
